# Lifetime prevalence of owner-reported medical conditions in the 25 most common dog breeds in the Dog Aging Project pack

**DOI:** 10.3389/fvets.2023.1140417

**Published:** 2023-11-03

**Authors:** Kiersten K. Forsyth, Brianah M. McCoy, Sarah M. Schmid, Daniel E. L. Promislow, Noah Snyder-Mackler, the DAP Consortium, Joshua M. Akey, Brooke Benton, Elhanan Borenstein, Marta G. Castelhano, Amanda E. Coleman, Kate E. Creevy, Kyle Crowder, Matthew D. Dunbar, Virginia R. Fajt, Annette L. Fitzpatrick, Unity Jeffery, Erica C Jonlin, Matt Kaeberlein, Elinor K. Karlsson, Kathleen F. Kerr, Jonathan M. Levine, Jing Ma, Robyn L McClelland, Daniel E. L. Promislow, Audrey Ruple, Stephen M. Schwartz, Sandi Shrager, Noah Snyder-Mackler, M. Katherine Tolbert, Silvan R. Urfer, Benjamin S. Wilfond, Kate E. Creevy

**Affiliations:** ^1^Department of Veterinary Clinical Sciences, College of Veterinary Medicine, Purdue University, West Lafayette, IN, United States; ^2^School of Life Sciences, Arizona State University, Tempe, AZ, United States; ^3^Department of Small Animal Clinical Sciences, College of Veterinary Medicine, University of Tennessee, Knoxville, TN, United States; ^4^Department of Laboratory Medicine and Pathology, University of Washington School of Medicine, Seattle, WA, United States; ^5^Department of Biology, University of Washington, Seattle, WA, United States; ^6^Center for Evolution and Medicine, Arizona State University, Tempe, AZ, United States; ^7^School for Human Evolution and Social Change, Arizona State University, Tempe, AZ, United States; ^8^Department of Small Animal Clinical Sciences, College of Veterinary Medicine & Biomedical Sciences, Texas A&M University, College Station, TX, United States

**Keywords:** lifetime prevalence, purebred, mixed-breed, epidemiology, cross-sectional

## Abstract

**Introduction:**

Large scale data on the prevalence of diverse medical conditions among dog breeds in the United States are sparse. This cross-sectional study sought to estimate the lifetime prevalence of medical conditions among US dogs and to determine whether purebred dogs have higher lifetime prevalence of specific medical conditions compared to mixed-breed dogs.

**Methods:**

Using owner-reported survey data collected through the Dog Aging Project (DAP) Health and Life Experience Survey for 27,541 companion dogs, we identified the 10 most commonly reported medical conditions in each of the 25 most common dog breeds within the DAP cohort. Lifetime prevalence estimates of these medical conditions were compared between mixed-breed and purebred populations. The frequency of dogs for whom no medical conditions were reported was also assessed within each breed and the overall mixed-breed and purebred populations.

**Results:**

A total of 53 medical conditions comprised the top 10 conditions for the 25 most popular breeds. The number of dogs for whom no medical conditions were reported was significantly different (p = 0.002) between purebred (22.3%) and mixed-breed dogs (20.7%). The medical conditions most frequently reported within the top 10 conditions across breeds were dental calculus (in 24 out of 25 breeds), dog bite (23/25), extracted teeth (21/25), osteoarthritis (15/25), and Giardia (15/25).

**Discussion:**

Purebred dogs in the DAP did not show higher lifetime prevalence of medical conditions compared to mixed-breed dogs, and a higher proportion of purebred dogs than mixed-breed dogs had no owner-reported medical conditions. Individual breeds may still show higher lifetime prevalence for specific conditions.

## 1. Introduction

The domestic dog (*Canis familiaris*) is a highly morphologically diverse species including various breeds developed over the past few hundred years resulting in dramatic phenotypic variations (20, 64). Humans have selected for specific phenotypic traits leading to the development of many pedigree dog breeds. There are currently 197 registered breeds with the American Kennel Club (AKC) and approximately 400 breeds recognized internationally (1). Inbreeding or line-breeding has been deployed in the development of most dog breeds, raising the concern that purebred dogs will have a greater chance of carrying genetic disorders (1, 12, 20). Inbreeding can lead to an increased prevalence of recessive genetic disorders, making what would normally be a rare disease more common within that breed or familial line (12, 20, 58); however, it can also lead to purging of the genetic load and associated elimination of inbreeding depression (10, 65). It is a common belief that purebred dogs are at a greater risk for disease compared to mixed-breed dogs, often due to concerns for genetic or inherited disorders; however, research has shown that this is not always the case. Some studies suggest specific breeds or groups of breeds are at greater risk for certain disorders, but simply being purebred may not necessarily be associated with increased disorder prevalence overall (43, 55).

The majority of studies evaluating breed predilections focus on either the breeds affected by a certain medical condition or the medical conditions most prevalent in a specific breed (32, 33, 37–39, 41, 42, 48, 60). For example, dilated cardiomyopathy has been determined to be far more common in Doberman Pinschers, Boxers, and Great Danes (32) than other dog breeds, whereas studies on the Cavalier King Charles Spaniel have found a high prevalence of cardiac, dermatological, and ocular disease (60). Comparatively, there are few studies that investigate the overall prevalence of disorders within and across breeds (21, 22, 66). Such studies are accomplished through either retrospective review of medical records or insurance data, or through health surveys completed by owners or veterinarians. Each of these methods of data acquisition have their own benefits and limitations.

Historically, prevalence data have been obtained via survey questionnaires distributed through breed clubs and associations; however, these surveys are often small-scale including only a few hundred to a few thousand dogs (12). With advancements in data sharing programs like VetCompass in the UK and Australia and the increased implementation of electronic medical record systems, large quantities of data can be collected and used to evaluate the demographic risk factors for various medical conditions (27, 33, 37–42, 44, 45, 50, 60). Pet insurance data has also been utilized to assess disease prevalence and mortality in the insured pet population (15). There are not currently any publicly accessible data sources like VetCompass in the US, making it difficult to perform a large-scale review of medical records to estimate prevalence of veterinary medical conditions in the US. Large-scale health surveys offer the chance to collect information from a more representative population, including those dogs who may not have seen a veterinarian within the timeframe of the study (66). The Dog Aging Project (DAP) is a nationwide community science research initiative; at its core is a long-term longitudinal study following companion dogs through annual surveys collecting biological, environmental, and lifestyle data.

This cross-sectional study aimed to estimate the lifetime prevalence of the most common owner-reported medical conditions (ORMC) for the top 25 breeds in the DAP, to identify differences in lifetime medical condition prevalence between the mixed-breed and purebred populations, and to identify differences in lifetime medical condition prevalence for the top 25 breeds when compared to other purebreds and to mixed-breed dogs. A secondary aim of this study was to determine whether or not there is a difference in prevalence of dogs with no ORMC between the purebred and mixed-breed populations.

## 2. Materials and methods

The DAP is a community science project in which owners enroll their companion dogs through a series of online surveys (8). The data collection instrument offered to all owners at the time of enrollment is a detailed Health and Life Experience Survey (HLES). All dogs whose owners complete HLES in its entirety become members of the DAP Pack for the duration of the study (or until the dog’s death or the owner drops out of the study). The DAP is an open data initiative with HLES data becoming available for analysis in yearly releases. The methodology of the DAP, including survey distribution and data collection, has been detailed previously (8). The University of Washington IRB deemed that recruitment of dog owners for the Dog Aging Project, and the administration and content of the DAP Health and Life Experience Survey (HLES), are human subjects research that qualifies for Category 2 exempt status (IRB ID no. 5988, effective 10/30/2018). No interactions between researchers and privately owned dogs occurred; therefore, IACUC oversight was not required. This cross-sectional study was performed using data collected from 27,541 dogs enrolled in the DAP Pack between December 26, 2019 and December 31, 2020.

HLES consists of 10 topical sections including owner and dog demographics, health status, diet, behavior, physical activity, and environmental factors. The full HLES questionnaire is publicly available for review online (13). Data from the Dog Demographics section of HLES were used to identify the 25 most common AKC-recognized breeds within the DAP Pack and to calculate descriptive statistics for the various breed groups. Data from the Health Status section of HLES were used to identify the top 10 ORMC for each of those 25 most common breeds. Because these data are collected directly from owners and because all medical problems may not have been fully investigated, term lists in the Health Status section include specific diagnoses, syndromes, and clinical signs; here we use the term “medical conditions” to embrace all of these findings. Within the Health Status section, owners are asked to report all medical conditions their dogs have experienced. Respondents select medical conditions within pathophysiologic (e.g., “Cancer or tumors” or “Infectious or parasitic disease”) and organ system [e.g., “Cardiac disorders” or “Kidney or urinary disorders” categories, and each category includes the option of “other (please describe)].” Free text responses recorded in these “other (please describe)” items were not included in the analysis of the medical conditions because this response choice does not represent a single medical condition and was therefore beyond the scope of this study. Free text categories for which the choice of “other (please describe)” would have ranked in the top 10 medical conditions for one or more of the 25 breeds include: other trauma, other oral condition, other eye condition, other orthopedic condition, and other skin condition. These “other (please describe)” free text responses will be coded and analyzed in a future study, and coded results will be made available in future data releases. For purposes of the study reported here, we do not attempt to refine, combine or otherwise modify participant responses, and present the data as reported by the participant. For instance, while it is plausible that a dog with “intervertebral disc disease” or “fibrocartilagenous embolism” also had “limb paralysis,” and vice versa, we report precisely what the participant indicated, without assuming that other related conditions were present.

### 2.1. Statistical analysis

Statistical analysis was performed using R software (version 4.1.1, R Foundation for Statistical Computing, Vienna, Austria). Dogs were separated into groups of mixed-breed dogs or purebred dogs based on owner-reported breed classification, which has been shown to be reliable (35). Here, we focused on the 25 most common AKC-recognized breeds within the DAP Pack. Descriptive statistics for dogs in each of the breeds were calculated including median age and median weight at the time survey data were collected. On average, small dogs live longer than large dogs, and often by a substantial margin. For this reason, simple chronological age is insufficient to describe the life stage (e.g., juvenile to adult to senior) in dogs of various sizes (18). Using the AAHA Canine Life Stage Guidelines (9), and the authors’ prior work on companion dog lifespan (62), average lifespan estimates were generated for groups of dog sizes in 10-kg increments, and life stages were assigned within size categories, as shown in Table 1. All dogs were assigned to a specific life stage based on age and body size. The mean and median life stage was determined for each of the 25 breeds and the mixed-breed category.

**Table 1:** Life stages by dog size.

Size	Puppy	Young adult	Mature adult	Senior
<10 kg	0–0.75 yr	> 0.75–3 yr	> 3–12 yr	> 12 yr
10–19.9 kg	0–0.75 yr	> 0.75–3 yr	> 3–12 yr	> 12 yr
20–29.9 kg	0–0.75 yr	> 0.75–3 yr	> 3–11 yr	> 11 yr
30–39.9 kg	0–1 yr	> 1.0–3 yr	> 3–10.5 yr	> 10.5 yr
≥ 40 kg	0–1.5 yr	> 1.5–3 yr	> 3–9.5 yr	> 9.5 yr

Within each of the 25 breeds, we calculated the lifetime prevalence of the 10 most commonly reported ORMC and generated 95% confidence intervals using the Wilson method. Lifetime prevalence estimates for these medical conditions were also calculated for all purebred, and all mixed-breed dogs, respectively, within the DAP Pack. We used a binomial model with logit link and adjusting for age with a linear effect to test: (1) whether mixed-breed and purebred dogs differed in lifetime medical condition prevalence and then (2) whether specific breeds exhibited statistically higher (or lower) lifetime prevalence of a given medical condition compared to all other purebred dogs in the dataset. The result of these models were age-adjusted log-odds ratios of medical conditions prevalence for a given breed compared to all other purebred dogs (or a comparison of mixed vs. purebred dogs). In the case where multiple statistical comparisons were carried out, we adjusted the threshold for significance using the Bonferroni correction. With 53 unique medical conditions (see below), this led to an adjusted significance threshold of *p* ≤ 0.05/53 = 0.00095 (e.g., Tables 2–5). When comparing the lifetime prevalence of dogs with no owner-reported medical conditions between the 25 breeds and mixed-breed dogs, this led to an adjusted significance threshold of *p* ≤ 0.05/25 = 0.002 (e.g., Table 6).

**Table 2:** Lifetime prevalence of 53 medical conditions in the purebred and mixed-breed dog populations.

	*N* (mixed-breed/purebred)	Mixed-breed Prevalence (%; *n* = 13,923)	Purebred Prevalence (%; *n* = 13,618)	*p*-value
No ORMC	2888/3031	20.74	22.26	**0.002**
Dental calculus	2089/2011	15.00	14.77	0.058
Extracted teeth	1877/810	13.48	5.95	**1.66 × 10**^**−98**^
Dog bite	1663/1305	11.94	9.58	**2.64 × 10**^**−10**^
Fractured teeth	997/810	7.16	5.95	**4.82 × 10**^**−5**^
Seasonal allergies	994/884	7.14	6.49	**0.033**
*Giardia*	952/1006	6.84	7.39	0.076
Osteoarthritis	908/869	6.52	6.38	0.636
Ear infection	860/1138	6.18	8.36	**3.11 × 10**^**−12**^
Torn or broken toenail	788/658	5.66	4.83	**0.002**
Chocolate ingestion	746/546	5.36	4.01	**1.21 × 10**^**−7**^
*Bordatella* and/or *parainfluenza*	719/558	5.16	4.10	**2.57 × 10**^**−5**^
Heart murmur	647/709	4.65	5.21	**0.032**
Pruritis	641/523	4.60	3.84	**0.002**
Laceration	540/433	3.88	3.18	**0.002**
Cruciate ligament rupture	535/447	3.84	3.28	**0.012**
Cataracts	527/626	3.79	4.60	**7.71 × 10**^**−4**^
Fleas	485/325	3.48	2.39	**7.17 × 10**^**−8**^
Sebaceous cysts	460/482	3.30	3.54	0.282
Food or medicine allergies that affect the skin	415/427	2.98	3.14	0.455
Gingivitis	414/475	2.97	3.49	**0.016**
Roundworms	412/366	2.96	2.69	0.174
Conjunctivitis	390/369	2.80	2.71	0.643
Hookworms	388/319	2.79	2.34	**0.020**
Chronic/recurrent hot spots	386/442	2.77	3.25	**0.022**
Lyme disease	377/333	2.71	2.45	0.169
Chronic/recurrent diarrhea	375/411	2.69	3.02	0.106
Patellar luxation	372/321	2.67	2.36	0.096
Anal sac impaction	353/367	2.54	2.69	0.407
Urinary tract infection (chronic/recurrent)	346/466	2.49	3.42	**4.32 × 10**^**−6**^
GI foreign body	329/357	2.36	2.62	0.169
Food or medicine allergies	309/337	2.22	2.47	0.162
Hip dysplasia	307/333	2.20	2.45	0.186
Hearing loss (incompletely deaf)	306/347	2.20	2.55	0.056
Urinary incontinence	303/332	2.18	2.44	0.148
Seizures	283/323	2.03	2.37	0.055
Pancreatitis	263/324	1.89	2.38	**0.005**
Underbite	252/115	1.81	0.84	**2.82 × 10**^**−12**^
Hypothyroidism	245/275	1.76	2.02	0.113
Lameness (chronic/recurrent)	240/240	1.72	1.76	0.807
Deafness	223/249	1.60	1.83	0.147
Urinary crystals or stones in bladder or urethra	194/228	1.39	1.67	0.058
Coccidia	184/254	1.32	1.87	**3.12 × 10**^**−4**^
Anaplasmosis	151/150	1.08	1.10	0.892
Fractured bone (limb)	146/170	1.05	1.25	0.120
Lick granuloma	144/142	1.03	1.04	0.945
Corneal ulcer	113/208	0.81	1.53	**3.14 × 10**^**−8**^
Intervertebral disc disease (IVDD)	112/206	0.80	1.51	**3.78 × 10**^**−8**^
Chronic kidney disease	109/105	0.78	0.77	0.911
Retained deciduous teeth	96/148	0.69	1.09	**4.35 × 10**^**−4**^
Keratoconjunctivitis sicca (KCS)	85/206	0.61	1.51	**2.46 × 10**^**−13**^
Tracheal collapse	76/93	0.55	0.68	0.145
Entropion	41/132	0.29	0.97	**1.37 × 10**^**−12**^
Stenotic/narrow nares	5/50	0.04	0.37	**7.44 × 10**^**−10**^

The bold values included in the tables are the *p*-values that are statistically significant.

**Table 3:** Lifetime prevalence of the top 10 medical conditions in Labrador Retrievers compared to the total purebred population.

Medical condition	Number affected	Lifetime Prevalence (%)	95% CI (%)	*p*-value
Ear Infection	226	13.51	11.95–15.23	**1.33 × 10**^**−16**^
Dog bite	159	9.50	8.19–11.00	0.836
Osteoarthritis	138	8.25	7.02–9.66	**7.42 × 10**^**−8**^
*Giardia*	126	7.53	6.36–8.90	0.912
Dental calculus	120	7.17	6.03–8.51	**2.99 × 10**^**−17**^
Seasonal allergies	120	7.17	6.03–8.51	0.170
Fractured teeth	119	7.11	5.98–8.45	0.006
Cruciate ligament rupture	115	6.87	5.76–8.19	**8.54 × 10**^**−20**^
Extracted teeth	102	6.10	5.05–7.35	**9.64 × 10**^**−20**^
*Bordetella* and/or parainfluenza (“kennel cough”)	91	5.44	4.45–6.63	0.004

The bold values included in the tables are the *p*-values that are statistically significant.

**Table 4:** Lifetime prevalence of the top 10 medical conditions in Golden Retrievers compared to the total purebred population.

Medical condition	Number affected	Lifetime Prevalence (%)	95% CI (%)	*p*-value
Ear Infection	179	12.23	10.65–14.00	**1.19 × 10**^**−10**^
*Giardia*	120	8.20	6.90–9.71	0.578
Chronic or recurrent hot spots	115	7.86	6.58–9.35	**3.67 × 10**^**−29**^
Dental calculus	103	7.04	5.83–8.46	**2.68 × 10**^**−10**^
Seasonal allergies	91	6.22	5.09–7.57	0.942
Dog bite	91	6.22	5.09–7.57	1.80 × 10^4^
Osteoarthritis	78	5.33	4.29–6.60	0.147
*Bordetella* and/or parainfluenza (“kennel cough”)	72	4.92	5.76–8.19	0.126
Fractured teeth	57	3.89	3.02–5.01	0.022
Pruritis (itchy skin)	57	3.89	3.02–5.01	0.640

The bold values included in the tables are the *p*-values that are statistically significant.

**Table 5:** Lifetime prevalence of the top 10 medical conditions in German Shepherd Dogs compared to the total purebred population.

Medical condition	Number affected	Lifetime Prevalence (%)	95% CI (%)	*p*-value
Seasonal allergies	63	10.36	8.18–13.04	**9.95 × 10**^**−6**^
Ear Infection	55	9.05	7.02–11.59	0.246
*Giardia*	53	8.72	6.73–11.23	0.421
Dog bite	51	8.39	6.44–10.86	0.783
Hip dysplasia	46	7.57	5.72–9.94	**9.76 × 10**^**−19**^
Osteoarthritis	43	7.07	5.29–9.39	5.95 × 10^−5^
Fractured teeth	39	6.41	4.73–8.65	0.085
Pruritis (itchy skin)	34	5.59	4.03–7.71	0.009
Sebaceous cysts	33	5.43	3.89–7.52	3.96 × 10^−5^
Torn or broken toenail	32	5.26	3.75–7.34	0.330

The bold values included in the tables are the *p*-values that are statistically significant.

**Table 6:** Lifetime prevalence of dogs with no owner-reported medical conditions (ORMC) in the total purebred dog population and the 25 most popular individual breeds compared to the mixed-breed dog population.

	No ORMC (*N*)	No ORMC (%)	95% CI	*p*-value
Purebred Dogs (all)	3,031	22.26%	21.57–22.96%	**0.002**
Mixed-Breed Dogs	2,888	20.74%	20.08–21.42%	
Labrador Retriever	378	22.59%	20.65–24.66%	0.079
Golden Retriever	367	25.07%	22.92–27.35%	**1.16×10**^**−4**^
German Shepherd Dog	150	24.67%	21.41–28.25%	0.020
Poodle	125	26.88%	23.05–31.09%	**0.001**
Australian Shepherd	126	29.17%	25.08–33.62%	**2.30 × 10**^**−5**^
Dachshund	72	21.11%	17.12–25.76%	0.867
Border Collie	94	31.54%	26.53–37.03%	**5.84 × 10**^**−6**^
Chihuahua	39	17.03%	12.72–22.43%	0.169
Beagle	41	19.90%	15.02–25.88%	0.768
Pembroke Welsh Corgi	53	26.50%	20.87–33.02%	0.047
Boxer	42	21.21%	16.09–27.43%	0.871
Shih Tzu	29	15.03%	10.67–20.75%	0.051
Miniature Schnauzer	39	20.31%	15.23–26.56%	0.884
Pug	30	15.96%	11.41–21.87%	0.108
Havanese	32	17.78%	12.88–24.02%	0.329
Cavalier King Charles Spaniel	41	24.12%	18.30–31.07%	0.281
Yorkshire Terrier	30	17.75%	12.73–24.21%	0.340
Great Dane	42	25.77%	19.67–32.98%	0.116
Greyhound	14	8.81%	5.32–14.24%	**2.15 × 10**^**−4**^
Boston Terrier	37	23.27%	17.38–30.42%	0.435
Siberian Husky	55	34.81%	27.82–42.52%	**1.53 × 10**^**−5**^
Shetland Sheepdog	33	21.43%	15.68–28.56%	0.835
English Springer Spaniel	35	23.33%	17.28–30.72%	0.437
Australian Cattle Dog	28	19.31%	13.71–26.49%	0.672
Doberman Pinscher	40	27.78%	21.11–35.60%	0.039

The bold values included in the tables are the *p*-values that are statistically significant.

## 3. Results

Data from 27,541 companion dogs were included in the study. Of those dogs, 50.6% were mixed-breed dogs (*n* = 13,923) and 49.4% were purebred dogs (*n* = 13,618). Of the purebred dogs, the 25 most commonly represented breeds are included in Table 7. These 25 breeds (*n* = 8,438) make up 62.0% of the purebred dog population and 30.6% of the overall DAP Pack. The remaining 38.0% of purebred dogs included members of 258 breeds. Demographic data describing these breed groups is summarized in Table 7. Both the mean and median life stage for all 25 breeds and the mixed-breed category fell into the mature adult life stage. The distribution of life stages within individual breeds was similar across the 25 breeds.

**Table 7:** Descriptive data for the DAP Pack including mixed-breed dogs, purebred dogs, and the 25 most popular individual breeds.

	Number of dogs	Age [years, mean (range)]	Life stage (mean)	Weight [kg, mean (range)]	Neutered Male [*n* (%)]	Intact Male [*n* (%)]	Spayed Female [*n* (%)]	Intact Female [*n* (%)]
Mixed-Breed Dogs	13,923	7.2 (0.1–24.8)	Mature adult	21.6 (<0.1–90.9)	6,593 (47.4)	222 (1.59)	6,982 (50.1)	162 (1.16)
Purebred Dogs (total)	13,618	7.4 (0.1–25.5)	Mature adult	24.0 (0.3–104.5)	5,852 (43.0)	1,147 (8.42)	5,991 (44.0)	628 (4.61)
Labrador Retriever	1,673	8.1 (0.2–24.8)	Mature adult	33.7 (4.2–59.1)	687 (41.1)	127 (7.59)	794 (47.5)	65 (3.89)
Golden Retriever	1,464	6.1 (0.3–19.4)	Mature adult	32.3 (7.0–57.7)	604 (41.3)	167 (11.41)	607 (41.5)	86 (5.87)
German Shepherd Dog	608	6.0 (0.3–19.4)	Mature adult	36.9 (14.7–67.3)	214 (35.2)	67 (11.02)	296 (48.7)	31 (5.1)
Poodle	465	7.2 (0.3–19.0)	Mature adult	20.1 (2.4–44.1)	230 (49.5)	38 (8.17)	183 (39.4)	14 (3.01)
Australian Shepherd	432	6.6 (0.2–17.7)	Mature adult	21.3 (3.2–44.1)	199 (46.1)	33 (7.64)	177 (41.0)	23 (5.32)
Dachshund	341	9.1 (0.2–20.8)	Mature adult	6.8 (1.8–18.2)	157 (46.0)	22 (6.45)	158 (46.3)	4 (1.17)
Border Collie	298	7.3 (0.3–17.0)	Mature adult	19.6 (10.6–42.7)	114 (38.3)	21 (7.05)	145 (48.7)	18 (6.04)
Chihuahua	229	9.7 (0.3–22.3)	Mature adult	3.7 (0.9–10.9)	109 (47.6)	5 (2.18)	104 (45.4)	11 (4.80)
Beagle	206	8.9 (0.6–21.0)	Mature adult	13.2 (6.4–25.0)	92 (44.7)	6 (2.91)	102 (49.5)	6 (2.91)
Pembroke Welsh Corgi	200	6.1 (0.3–20.3)	Mature adult	13.1 (4.5–21.4)	91 (45.5)	9 (4.50)	91 (45.5)	9 (4.5)
Boxer	198	6.3 (0.5–20.8)	Mature adult	29.2 (16.8–44.5)	89 (44.9)	11 (5.56)	90 (45.5)	8 (4.04)
Shih Tzu	193	9.4 (0.3–18.0)	Mature adult	6.5 (2.3–14.5)	106 (54.9)	11 (5.70)	73 (37.8)	3 (1.55)
Miniature Schnauzer	192	7.9 (0.3–17.8)	Mature adult	8.0 (3.0–15.5)	85 (44.3)	9 (4.69)	95 (49.5)	3 (1.56)
Pug	188	8.8 (0.3–17.3)	Mature adult	9.5 (4.7–17.3)	93 (49.5)	1 (0.53)	90 (47.9)	4 (2.13)
Havanese	180	7.7 (0.4–19.2)	Mature adult	6.6 (2.7–13.6)	92 (52.4)	5 (2.78)	78 (43.3)	5 (2.78)
Cavalier King Charles Spaniel	170	7.1 (0.3–14.9)	Mature adult	9.1 (2.7–17.7)	89 (52.4)	7 (4.12)	70 (41.2)	4 (2.35)
Yorkshire Terrier	169	9.4 (0.9–22.0)	Mature adult	3.9 (1.4–9.8)	80 (47.3)	4 (2.37)	82 (48.5)	3 (1.78)
Great Dane	163	5.1 (0.3–12.2)	Mature adult	59.6 (22.7–88.6)	55 (33.7)	21 (12.88)	79 (48.5)	8 (4.91)
Greyhound	159	7.5 (1.8–14.2)	Mature adult	31.8 (21.4–45.5)	78 (49.1)	2 (1.26)	77 (48.4)	2 (1.26)
Boston Terrier	159	7.2 (0.1–16.0)	Mature adult	9.9 (2.3–20.0)	70 (44.0)	7 (4.40)	75 (47.2)	7 (4.4)
Siberian Husky	158	7.2 (0.7–19.3)	Mature adult	25.4 (13.2–45.9)	65 (41.1)	7 (4.43)	80 (50.6)	6 (3.8)
Shetland Sheepdog	154	8.0 (0.5–19.0)	Mature adult	12.0 (5.5–22.7)	81 (52.6)	11 (7.14)	58 (37.7)	4 (2.6)
English Springer Spaniel	150	7.6 (0.4–16.7)	Mature adult	21.1 (10.5–35.5)	52 (34.7)	4 (2.67)	85 (56.7)	9 (6.0)
Australian Cattle Dog	145	7.4 (0.3–18.5)	Mature adult	21.2 (11.8–33.6)	64 (44.1)	5 (3.45)	71 (49.0)	5 (3.45)
Doberman Pinscher	144	6.1 (0.4–13.6)	Mature adult	34.5 (5.0–54.5)	55 (38.2)	15 (10.42)	67 (46.5)	7 (4.86)

**Figure 1:**
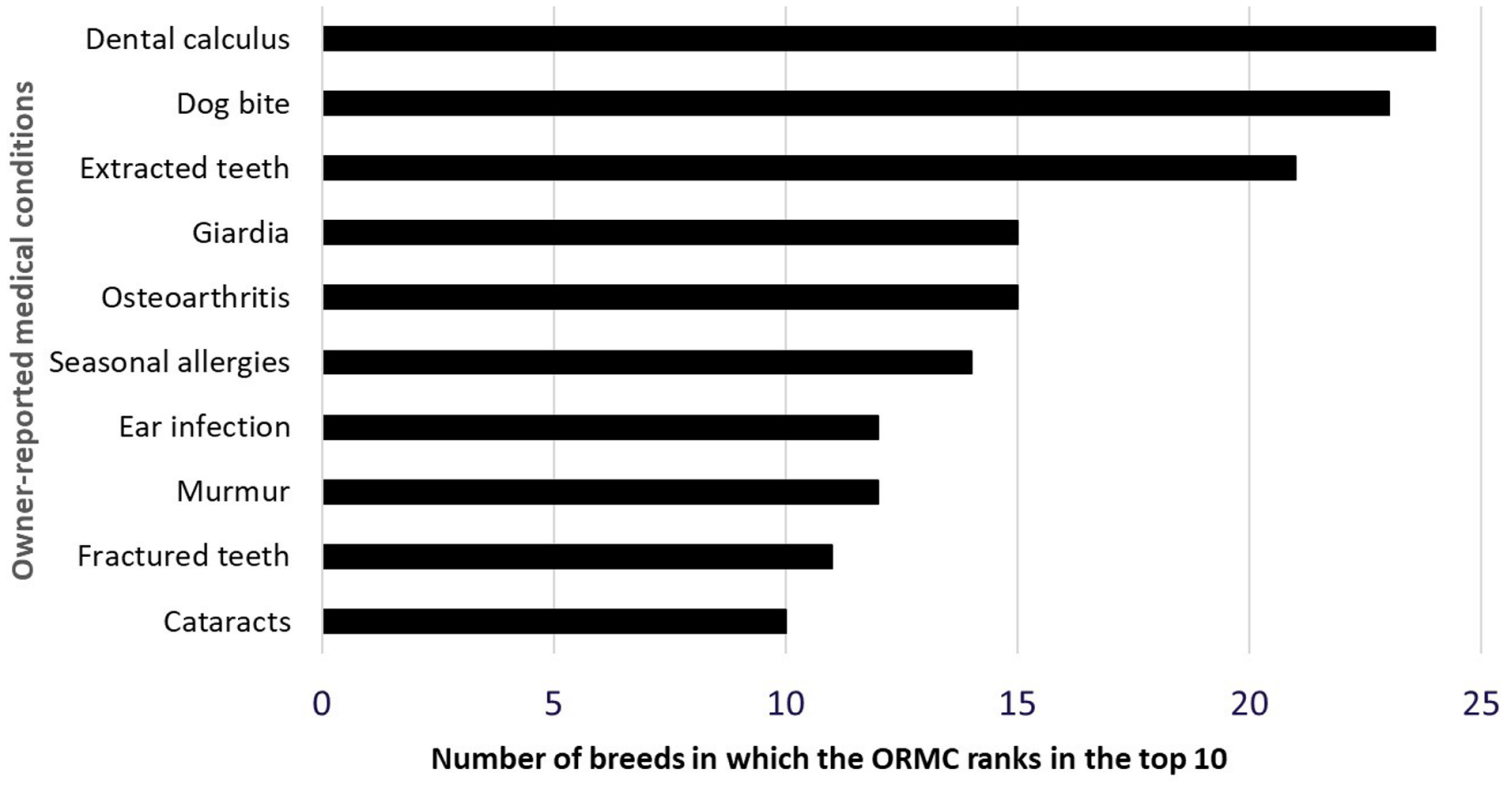
Medical conditions which rank in the top 10 medical conditions in at least 10 of the 25 most popular individual dog breeds in the DAP Pack.

**Figure 2:**
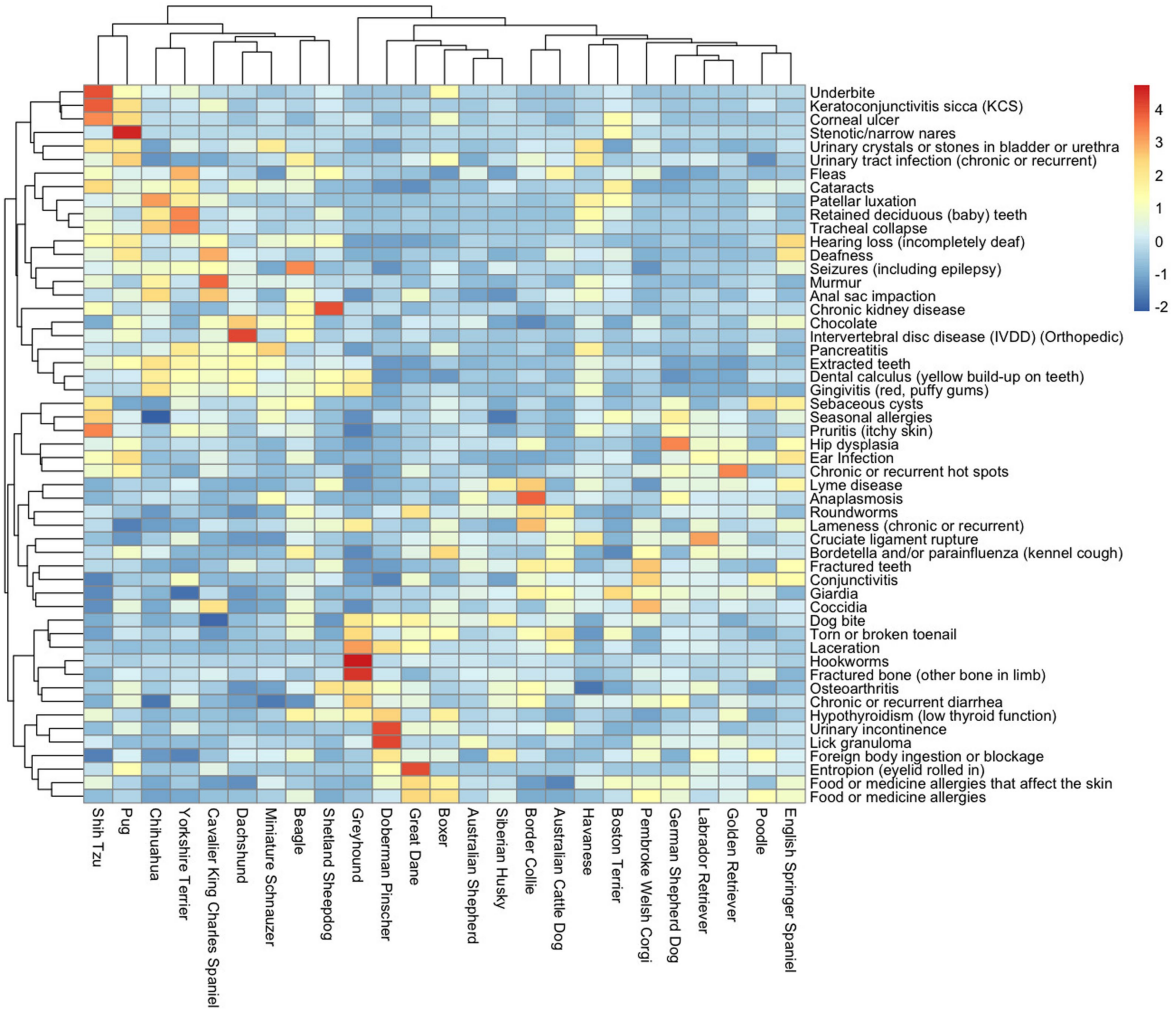
Heatmap showing the scaled lifetime prevalence of the 53 medical conditions (vertical axis) within the 25 most popular individual breeds (horizontal axis) in the DAP pack, with medical condition and breed clustered by similarity. The red color denotes higher lifetime prevalence, while the blue color denotes lower lifetime prevalence.

A total of 53 unique medical conditions were identified making up the top 10 ORMC for these 25 breeds. The top 10 ORMC in mixed-breed dogs were a subset of these 53 medical conditions. The ORMC reported most frequently across breeds were dental calculus, dog bites, extracted teeth, osteoarthritis, and *Giardia* (Figure 1). Data on all 53 medical conditions and their lifetime prevalence in individual breeds are available in Supplementary Table 1. Data on the top 10 ORMC and their lifetime prevalence in individual breeds are available as Supplementary Table 2. A Heatmap (Figure 2) demonstrates the scaled prevalence of these 53 medical conditions across the 25 most popular dog breeds in the DAP Pack.

### 3.1. Dogs with no reported medical conditions

Purebred dogs (22.3%) were significantly more likely (*p* = 0.002) to have no ORMC than mixed-breed dogs (20.7%). Considering each breed compared to the population of mixed-breed dogs, significantly more Golden Retrievers (25.1%; *p* = 1.16 × 10^−4^), Poodles (26.9%; *p* = 0.001), Australian Shepherds (29.2%; *p* = 2.3 × 10^−5^), Border Collies (31.5%; *p* = 5.84 × 10^−6^), and Siberian Huskies (34.8%; *p* = 1.53 × 10^−5^) had no ORMC compared to mixed-breed dogs (Table 6). Conversely, the frequency of Greyhounds with no ORMC was significantly lower (8.8%; *p* = 2.15 × 10^−4^) than mixed-breed dogs.

### 3.2. Overall purebred vs. mixed-breed results

A total of 14 ORMC were significantly more prevalent in the overall purebred dog population compared to the overall mixed-breed dog population, and similarly, 13 ORMC were significantly more prevalent in mixed-breed dogs compared to purebred dogs (Table 2). The remaining 26 ORMC were not significantly different between the mixed-breed and purebred dog populations. The most common ORMC in mixed-breed dogs were dental calculus, extracted teeth, dog bite, seasonal allergies, fractured teeth, giardia, osteoarthritis, ear infection, torn or broken toenail, and chocolate toxicity (Table 2).

### 3.3. Breed specific results

Data on the eight of the most popular breeds are described in detail here; data for the remaining 17 breeds in the top 25 are available in Supplementary Table 2. Additional information including the lifetime prevalence of the other ORMC within the union set of 53 medical conditions that did not rank in the top 10 ORMC within each breed are available in Supplementary Table 1. The top ORMC in Labrador Retrievers were ear infections, dog bites, osteoarthritis, *Giardia*, dental calculus, seasonal allergies, fractured teeth, cruciate ligament rupture, extracted teeth, and *Bordetella* and/or parainfluenza (Table 3). Labrador Retrievers were significantly more likely to be reported to have had ear infections (logOR = 0.66, *p* = 1.33 × 10^−16^), osteoarthritis (logOR = 0.55, *p* = 7.42 × 10^−8^), and cruciate ligament rupture (logOR = 1.03, *p* = 8.54 × 10^−20^) compared to other purebred dogs. Labrador Retrievers were significantly less likely to be reported to have dental calculus (logOR = −0.85, *p* = 2.99 × 10^−17^) and extracted teeth (logOR = −0.99, *p* = 9.64 × 10^−20^) compared to other purebred dogs.

The top ORMC in Golden Retrievers were ear infections, *Giardia*, chronic/recurrent hot spots, dental calculus, seasonal allergies, dog bites, osteoarthritis, *Bordetella* and/or parainfluenza, fractured teeth, and pruritis (Table 4). Golden Retrievers were significantly more likely to be reported to have ear infections (logOR = 0.56, *p* = 1.19 × 10^−10^) and chronic/ recurrent hot spots (logOR = 1.28, *p* = 3.67 × 10^−29^) compared to the overall purebred population. Golden Retrievers were significantly less likely to be reported to have dental calculus (logOR = −0.68,*p* = 2.68 × 10^−10^) compared to other purebred dogs.

The top ORMC in German Shepherd Dogs were seasonal allergies, ear infections, *Giardia*, dog bites, hip dysplasia, osteoarthritis, fractured teeth, pruritis, sebaceous cysts, and torn or broken toenails (Table 5). German Shepherd Dogs were significantly more likely to be reported to have hip dysplasia (logOR = 1.48, *p* = 9.76 × 10^−19^) and seasonal allergies (logOR = 0.61, *p* = 9.95 × 10^−6^) than other purebred dogs. The lifetime prevalence of the remaining eight ORMC in German Shepherd Dogs were not significantly different compared to other purebred dogs.

Compared to the overall purebred population, the lifetime prevalence of the top 10 ORMC in Australian Shepherds, Australian Cattle Dogs, Boxers, and Siberian Huskies were not significantly different from other purebred dogs (Supplementary Table 1). Both Australian Shepherds and Siberian Huskies were also found to have a significantly higher percentage of dogs with no ORMC than the mixed-breed population (Table 6).

Four of the 10 most commonly reported ORMC in Chihuahuas involved dental conditions: extracted teeth, dental calculus, gingivitis, and retained deciduous teeth (Supplementary Table 2). Specifically, 47.60% (41.22–54.05%) of Chihuahuas were reported to have had teeth extracted and 33.62% (27.82–39.97%) had dental calculus reported. This is significantly more frequently than either of those ORMC were reported in the purebred population (*p* = 6.12 × 10^−22^ and *p* = 4.52 × 10^−6^, respectively).

## 4. Discussion

The primary objective of this study was to describe the medical conditions most commonly reported by owners of the most popular breeds in the DAP Pack. Although there was the potential for 250 unique ORMC when looking at the 10 most common conditions for each of the 25 breeds, only 53 ORMC were identified. While the majority of these ORMC only placed in the top 10 for one or two of the 25 breeds, 10 of the ORMC were identified as highly common among at least 10 of the 25 breeds.

Dental calculus was among the top 10 ORMC in 24 of the 25 breeds, and extracted teeth were among the top 10 ORMC in 21 of the 25 breeds. This shows that dental conditions and dental care are relatively common across breeds in the US. Although dental conditions are seen across breeds, certain breeds are impacted more greatly, like Chihuahuas, for whom owners reported dental calculus in approximately one-third of breed members in the DAP Pack and extracted teeth in nearly half the breed members. O’Neill and others reported a 19% prevalence of dental disease among the Chihuahua population in the UK (41); it is possible that the difference is attributable to the fact that their study was a period prevalence estimate for the year 2016, whereas our study considers lifetime prevalence. A 1999 study demonstrated a prevalence of dental calculus at 20.5% as reported by veterinarians for dogs attending private small animal practices in the US (30). This is a higher rate of dental calculus for dogs overall than was noted in our study. This difference may be due to changes in prevalence and improved dental care and prophylaxis over the past 20 years, or may be due to a difference between veterinarian-reported and owner-reported information. Additional studies have shown that dental disorders are one of the most common medical conditions amongst various breeds in the UK (40, 45) and US (17), and dental conditions are a priority area of interest for future health-related welfare improvement in the UK dog population (59), particularly considering that regular veterinary dental scaling is associated with longer lifespan in dogs (63).

Interestingly, dog bite injuries were the second most common ORMC reported across breeds, identified in 23 of the top 25 breeds in the DAP Pack. Most prevalence data for dog bite injuries report on humans being bitten, with scarce data on dogs being bitten by other dogs (5, 23, 29, 49, 61). As a result, there are no recent prevalence data for dog-on-dog bite injuries in the US. The 23 affected breeds included dogs of all body sizes, from toy and small breeds to large and giant breed dogs. While owners and veterinarians may have concern that small dogs may be bitten by larger dogs, this study shows that bite injuries are quite common regardless of size. This highlights the importance of client education along with training and early socialization of dogs, to try to reduce the incidence of bite wounds in the future. Owner-reported dog bite injuries were more common in our study than previously reported in other studies (30); in fact, most prior studies did not even include dog bite injuries or animal bite injuries specifically in their analyses (17, 27, 33, 37–42, 45, 60, 66). It may be that dog bite injuries are being included in more broad categories such as trauma in other studies. Alternatively, it is possible that dog bite injuries are being overlooked in breed-related disease prevalence studies, as trauma of any kind may not be considered a form of disease. The study reported here described individual medical conditions, rather than overarching categories such as “trauma.” Within the category of trauma, conditions that occurred within the top 10 most frequent ORMC for any of the top 25 breeds were dog bite wound, torn or broken toenail, laceration and fractured bone. Other factors, such as lifestyle and environment, likely contribute to risk of medical conditions among dogs, and perhaps more significantly for traumatic conditions. However, it is likely that breed remains a significant risk factor for some traumatic conditions. For instance, certain breeds may be inherently more active, or may be disproportionately chosen by owners for active lifestyles; in either case, breed and environment could interact to increase the risk of traumatic injury. Future studies can further investigate these interrelationships.

The secondary objective of this study was to investigate whether or not there is a difference between purebred or mixed-breed dogs in lifetime prevalence of ORMC, or dogs with no ORMC. This study showed that the lifetime prevalence of dogs with no ORMC was higher in the purebred population (22.3%) compared to the mixed-breed population (20.7%) in the DAP Pack. This is contrary to the common belief that purebred dogs have a greater risk for developing medical conditions due to breed predispositions. This is also a higher proportion of dogs with no medical conditions than the 6.8% reported in the previously mentioned study which used veterinarian-reported data from small animal practices (30). It is possible that the difference between the two studies’ findings reflects the fact that, despite the survey instructions to report all known medical conditions, owners may not recall or choose to report all of their dogs’ medical conditions in the survey-based data used here. Alternatively, it is possible that the DAP Pack captures data from dogs from a general population of healthy dogs who do not require or receive veterinary care and therefore are not represented in manuscripts reporting only data derived from veterinary practice records. This intriguing possibility merits further investigation by determining the frequency of veterinary visits among DAP Pack members, and by comparing owner-reported data to data captured in medical records; such analyses are currently underway by the DAP. Although the difference between dogs with no ORMC in the purebred and mixed-breed populations was statistically significant, the difference was only 1.5%. The mixed-breed population had a significantly higher lifetime prevalence than the purebred population for 13 of the 53 ORMC described here; similarly, the purebred population had a significantly higher lifetime prevalence than the mixed-breed population for 14 of the 53 ORMC as well. Additionally, our study revealed that certain breeds were shown to have a higher lifetime prevalence of specific medical conditions. Overall, 23 of the 25 breeds evaluated showed a significantly increased lifetime prevalence of at least one ORMC when compared to other purebred dogs. Of the breeds evaluated, only the Australian Shepherd, Australian Cattle Dog, Boxer, and Siberian Husky showed no significant difference in lifetime prevalence for their 10 most commonly reported medical conditions when compared to other purebred dogs. Even when evaluating for lifetime prevalence in all 53 of the included ORMC, there was no significant difference in lifetime prevalence for the Australian Cattle Dog and Siberian Husky compared to other purebred dogs (Supplementary Table 1). Similarly, the Australian Shepherd and Siberian Husky were the breeds with the highest frequency of dogs with no ORMC in our study and significantly more dogs from these breeds had no ORMC compared to the mixed-breed population. Because the data utilized in this study are owner-reported and individual medical records associated with each dog were not evaluated as part of this study, we are unable to determine the severity of the ORMC or how that severity might vary between breeds. Future studies of severity of disease among breeds, and response to therapies deployed, are important targets of this research.

The most common breeds and ORMC seen in the DAP Pack are likely to be representative of the United States canine population. Most previous studies evaluating dogs within the US have been performed at veterinary referral centers (6, 16, 54) or within a single large private-practice chain (44, 50, 55). This creates a bias as these studies do not include dogs whose owners are unable to or elect not to pursue veterinary specialty referral (3), or target only a particular consumer group who may not be representative of the overall population. Because the dogs within the DAP Pack are recruited directly from owners, not through veterinarians or veterinary clinics, there is the possibility to include dogs who are evaluated by veterinarians less frequently, who are never seen at veterinary referral centers, and potentially those who have never seen a veterinarian at all. Any owner within the US can sign up at any time. While there is currently a trend towards older dogs and older owners within the DAP Pack, the DAP is actively recruiting for dogs of all ages and owners from more varying backgrounds. There is also a trend towards dogs which have been neutered within the DAP Pack. Because the majority of dogs within the DAP Pack are neutered, sex, neuter status, and age of neutering have not been assessed within this study. This is a limitation of this study, as these factors can influence medical conditions (21, 22).

The top 25 breeds making up the DAP Pack vary somewhat when compared to the top 25 breeds according to AKC registration numbers for 2020. Border Collie, Chihuahua, Pug, Greyhound, Shetland Sheepdog, and English Springer Spaniel all ranked higher in the DAP Pack than they did in AKC registration, as they were not even included in the AKC top 25 breeds (2). These differences between the reported AKC numbers and DAP Pack numbers are likely due to the fact that there are many purebred dogs in the US that are never registered with the AKC. Greyhounds are an excellent example of this as many of the Greyhounds in the DAP Pack are retired racing dogs, which are often registered with the National Greyhound Association instead of the AKC (36). It is also possible that this is the reason our study found significantly fewer greyhounds with no ORMC, as many racing greyhounds are retired due to injuries. However, despite these differences there are also similarities between popular DAP Pack and AKC breeds, such as the top 3 breeds in the DAP Pack ranking within the top 4 breeds in the AKC (2). This study classified all mixed-breed dogs as part of the mixed-breed population, including new “designer breeds” such as goldendoodles or cockapoos. While designer breeds are actually F1 hybrids and not purebred dog breeds, many are becoming popular within the United States. As a result, performing a similar study evaluating F1 hybrids as a third breed-background group could provide useful information for practicing veterinarians in the United States.

One of the main limitations of this study is that the data were collected through owner-reported survey results, meaning there is an inherent risk of recall bias and reporting bias affecting results. This may be because of lack of understanding of one or more conditions, lack of recollection of one or more condition and/or lack of comprehensive diagnostic investigation of one or more conditions. With these limitations in mind, our survey was intentionally designed to include clinical signs, as well as discrete diagnoses, among the options presented to participants. Additionally, data were intentionally analyzed as reported, and no attempt was made to interpret responses as components of a larger disease syndrome, or to assume the presence of common comorbidities. No previous large studies have been performed analyzing the accuracy of owner-reported medical data in veterinary medicine. Further research comparing owner-reported survey data to veterinary medical records is needed. Studies in human pediatrics show reasonable accuracy in parental recall of children’s health information, with greater accuracy for acute and significant health events (4, 11, 14, 19, 24, 34, 51–53, 57). Assuming that dog owners will have similar recall accuracy and biases as parents completing surveys about their children’s health, it is reasonable to assume that owner-reported surveys will be relatively accurate to their dog’s medical conditions with a potential bias towards recalling medical conditions which owners regard as more significant. As a result, this study may highlight those medical conditions that owners most recall or are most concerned about for their dogs. This means that there may be an underrepresentation of medical conditions that owners do not find concerning, or a lack of recognition of all components of a given medical syndrome by some respondents. An example of this is the low lifetime prevalence of stenotic nares, underbite, and tracheal collapse in this study. These three conditions were each reported in less than 1% of the purebred population and less than 2% of the mixed-breed population, much less frequent than reported in previous studies (25, 28, 31). Stenotic nares and underbite are often associated with brachycephalic breeds and may potentially be considered a breed standard in some, and as a result may not be recognized as a medical condition by owners or veterinarians (28, 46, 47). Furthermore, dogs with externally apparent brachycephalic features often have hypoplastic trachea as well (7, 26, 56), which less likely to be apparent to an owner, and may be under-reported in our data. We did not attempt to re-classify reports of external brachycephalic features into an overarching diagnosis of “brachycephalic syndrome” that assumed hypoplastic trachea was also present. Additional studies comparing ORMC to what is reported in the medical records are indicated to determine the accuracy of survey data in veterinary medicine.

Another limitation is the risk that owners will only recall the most recent medical conditions, resulting in underreporting of congenital disorders or medical conditions more commonly associated with young age. While dogs of all ages were included in this study, the DAP Pack is predominantly older dogs with the median ages of the different breeds ranging from 5.1 years (Great Dane) to 9.7 years (Chihuahua). All the breeds included in this study and the mixed-breed category had a majority of the participating dogs in the mature adult life stage. If owners do fail to recall medical conditions that were further in the past, the median age of the DAP Pack population may lead to a reporting towards medical conditions more common in older dogs. However, since the median life stages are similar among breeds in the DAP Pack, there should be minimal age-related biases in direct breed-to-breed comparisons. Furthermore, as the DAP is a longitudinal study, ORMC will be collected each year which will make it possible to construct a more accurate description of the timing of ORMC acquisition across the lifespan.

Another limitation of this study is that sex, neuter status, and age of neutering were not assessed in relation to the ORMC.

Overall, our study found that purebred dogs did not have an increased lifetime prevalence of ORMC compared to mixed-breed dogs; in fact the frequency of dogs with no ORMC was higher within the purebred population. However, specific breeds often show an increased lifetime prevalence of certain medical conditions. As a result, it is important for veterinarians to consider potential breed predispositions when monitoring and treating their patients. This study also revealed that dental conditions and dog bites are common across breeds, and as a result prevention for these conditions should be addressed by primary care veterinarians.

## Data availability statement

The datasets presented in this study can be found in online repositories. The names of the repository/repositories and accession number(s) can be found at: https://dogagingproject.org/open_data_access/.

## Ethics statement

The University of Washington IRB deemed that recruitment of dog owners for the Dog Aging Project, and the administration and content of the DAP Health and Life Experience Survey (HLES), are human subjects research that qualifies for Category 2 exempt status (IRB ID no. 5988, effective 10/30/2018). No interactions between researchers and privately owned dogs occurred; therefore, IACUC oversight was not required. The studies were conducted in accordance with the local legislation and institutional requirements. The participants provided their written informed consent to participate in this study.

## Author contributions

The DAP Consortium designed the DAP study, implemented data collection, and developed and curated the DAP databases. KKF, KEC, and SS designed the specific study. KKF, BMM, NS-M, and DELP completed the analyses and made the figures. KKF wrote the first draft of the manuscript. All authors contributed to the article and approved the submitted version.

## DAP Consortium

Joshua M. Akey^1^, Brooke Benton^2^, Elhanan Borenstein^3,4,5^, Marta G. Castelhano^6,7^, Amanda E. Coleman^8^, Kate E. Creevy^9^, Kyle Crowder^10,11^, Matthew D. Dunbar^11^, Virginia R. Fajt^12^, Annette L. Fitzpatrick^13,14,15^, Unity Jeffery^16^, Erica C Jonlin^2,17^, Matt Kaeberlein^2^, Elinor K. Karlsson^18,19^, Kathleen F. Kerr^20^, Jonathan M. Levine^9^, Jing Ma^21^, Robyn L McClelland^20^, Daniel E. L. Promislow^2,22^, Audrey Ruple^23^, Stephen M. Schwartz^24,14^, Sandi Shrager^25^, Noah Snyder-Mackler^26,27,28^, M. Katherine Tolbert^9^, Silvan R. Urfer^2^, Benjamin S. Wilfond^29,30^

^1^Lewis-Sigler Institute for Integrative Genomics, Princeton University, Princeton, NJ, United States

^2^Department of Laboratory Medicine and Pathology, University of Washington School of Medicine, Seattle, WA, United States

^3^Department of Clinical Microbiology and Immunology, Sackler Faculty of Medicine, Tel Aviv University, Tel Aviv, Israel

^4^Blavatnik School of Computer Science, Tel Aviv University, Tel Aviv, Israel

^5^Santa Fe Institute, Santa Fe, NM, United States

^6^Cornell Veterinary Biobank, College of Veterinary Medicine, Cornell University, Ithaca, NY, United States

^7^Department of Clinical Sciences, College of Veterinary Medicine, Cornell University, Ithaca, NY, United States

^8^Department of Small Animal Medicine and Surgery, College of Veterinary Medicine, University of Georgia, Athens, GA, United States

^9^Department of Small Animal Clinical Sciences, Texas A&M University College of Veterinary Medicine & Biomedical Sciences, College Station, TX, United States

^10^Department of Sociology, University of Washington, Seattle, WA, United States

^11^Center for Studies in Demography and Ecology, University of Washington, Seattle, WA, United States

^12^Department of Veterinary Physiology and Pharmacology, Texas A&M University College of Veterinary Medicine and Biomedical Sciences, College Station, TX, United States

^13^Department of Family Medicine, University of Washington, Seattle, WA, United States

^14^Department of Epidemiology, University of Washington, Seattle, WA, United States

^15^Department of Global Health, University of Washington, Seattle, WA, United States

^16^Department of Veterinary Pathobiology, Texas A&M University College of Veterinary Medicine & Biomedical Sciences, College Station, TX, United States

^17^Institute for Stem Cell and Regenerative Medicine, University of Washington, Seattle, WA, United States

^18^Bioinformatics and Integrative Biology, University of Massachusetts Chan Medical School, Worcester, MA, United States

^19^Broad Institute of MIT and Harvard, Cambridge, MA, United States

^20^Department of Biostatistics, University of Washington, Seattle, WA, United States

^21^Division of Public Health Sciences, Fred Hutchinson Cancer Research Center, Seattle, WA, United States

^22^Department of Biology, University of Washington, Seattle, WA, United States

^23^Department of Population Health Sciences, Virginia-Maryland College of Veterinary Medicine, Virginia Tech, Blacksburg, VA, United States

^24^Epidemiology Program, Fred Hutchinson Cancer Research Center, Seattle, WA, United States

^25^Collaborative Health Studies Coordinating Center, Department of Biostatistics, University of Washington, Seattle, WA, United States

^26^School of Life Sciences, Arizona State University, Tempe, AZ, United States

^27^Center for Evolution and Medicine, Arizona State University, Tempe, AZ, United States

^28^School for Human Evolution and Social Change, Arizona State University, Tempe, AZ, United States

^29^Treuman Katz Center for Pediatric Bioethics, Seattle Children’s Research Institute, Seattle, WA, United States

^30^Department of Pediatrics, Division of Bioethics and Palliative Care, University of Washington School of Medicine, Seattle, WA, United States
